# Oscillatory entrainment to our early social or physical environment and the emergence of volitional control

**DOI:** 10.1016/j.dcn.2022.101102

**Published:** 2022-03-25

**Authors:** S.V. Wass, M. Perapoch Amadó, J. Ives

**Affiliations:** Department of Psychology, University of East London, UK

**Keywords:** Synchrony, Entrainment sensitivity, Contingent responsiveness, Oscillations, Effortful control, Executive control, Infancy

## Abstract

An individual’s early interactions with their environment are thought to be largely passive; through the early years, the capacity for volitional control develops. Here, we consider: how is the emergence of volitional control characterised by changes in the entrainment observed between internal activity (behaviour, physiology and brain activity) and the sights and sounds in our everyday environment (physical and social)? We differentiate between contingent responsiveness (entrainment driven by evoked responses to external events) and oscillatory entrainment (driven by internal oscillators becoming temporally aligned with external oscillators). We conclude that ample evidence suggests that children show behavioural, physiological and neural entrainment to their physical and social environment, irrespective of volitional attention control; however, evidence for oscillatory entrainment beyond contingent responsiveness is currently lacking. Evidence for how oscillatory entrainment changes over developmental time is also lacking. Finally, we suggest a mechanism through which periodic environmental rhythms might facilitate both sensory processing and the development of volitional control even in the absence of oscillatory entrainment.

## 1 Introduction

All the laws are in perfect harmony with the aspirations of the mind. (Maharishi Mahesh; Yogi, 1968).

### 1.1 The ghost and the machine

The capacity for volitional control over attention is generally thought to be largely absent at birth, and to emerge gradually during the first few years of life (Colombo and Cheatham, 2006, Davidson et al., 2006, Hendry et al., 2016, Mundy and Newell, 2007, Wass, 2020) (although see (Emberson et al., 2015; Hodel, 2018)). During the first few months of life, infants’ orienting of visual attention is thought to be driven largely by whatever in their environment is most salient (i.e., automatically attention-eliciting) (Bronson, 1990, Johnson, 1990). As they develop, they become more active agents, with greater capacity to volitionally reorient attention (Colombo and Cheatham, 2006, Kulke et al., 2017; Smith et al., 2017) and to initiate social exchanges (Mundy and Newell, 2007). To borrow a concept from Descartes expressed in the terms of Gilbert Ryle, gradually during the first few years of life the ‘ghost’ (the active, volitional component of cognition) learns to ‘drive the machine’ (Ryle, 1949).

Recent research has made strides in understanding how the emergence of the capacity for active, volitional control is characterised by changes in how different systems *within* an individual (behaviour, physiology and brain) inter-relate – known as *intra*-individual entrainment (Grayson and Fair, 2017, Helfrich and Knight, 2016, Johnson, 2015, Munakata et al., 2011, Shine et al., 2016). Here, we consider a different question: how is the emergence of volitional control characterised by changes in the inter-relationship between the activity within an individual (i.e. behaviour, physiology and neural activity) and the environment in which that individual is located (i.e. sights and sounds originating from objects and people around us)?

### 1.2 Two types of entrainment

How do we quantify this inter-relationship? Here, we focus on entrainment: the similarity between two patterns of activity which change over time (see Section 2.2 for formal definitions). Entrainment is more commonly studied within an individual, to look either at the associations between behaviours such as vocalisations and autonomic activity (Y. S. Zhang and Ghazanfar, 2020) (see also Section 2), or between physiological and neural systems (Breeden et al., 2016), or (particularly) between discrete areas within a single individual’s brain (Grayson and Fair, 2017, Helfrich and Knight, 2016). Here we concentrate on a different aspect, namely entrainment between oscillations within an individual (either behaviour, physiology and neural activity) and specific aspects of their environment.

We shall differentiate between two types of entrainment. The first, which we shall call contingent responding, is used to describe responses evoked within an individual to changes in their outside environment – whether or not those changes are expected or unexpected, and periodic or aperiodic. (These two terms are not coreferential (Jaffe et al., 2001, Rimmele et al., 2018).) The second, which we shall call oscillatory entrainment, is more specific: it refers to already existent periodic activity patterns (oscillations) within an individual becoming coupled, or temporally aligned, with oscillations in the environment (Haegens, 2020, Haegens and Golumbic, 2018, Meyer et al., 2020a, Meyer et al., 2020b, Rosenblum et al., 2000).

Mechanistically, in terms both of how they are substantiated and of their potential benefits or functions in information processing, contingent responding and oscillatory entrainment are completely different, as we shall see in Section 2. The majority of the discussion into entrainment concentrates on the latter – treating all rhythms as if they are periodic, which they are not. In fact, as we shall argue, telling the two apart is harder than it appears. For example, if we present a regular pulse at 4 Hz and find that brain activity is also observed at 4 Hz, and if we then speed up the pulse to 5 Hz and observe that the dominant frequency of brain activity increases to 5 Hz, then we might conclude (as some have) that this is evidence for oscillatory entrainment to the pulses. In fact, though, such a finding would equally be expected based on a contingent responding model (see Section 2.2.1) (Capilla et al., 2011, Haegens and Golumbic, 2018, Meyer et al., 2020a).

### 1.3 Summary of what is to come

The aim of the article is to examine these different types of entrainment – concentrating in particular on early-life oscillatory entrainment, which has received little discussion hitherto (although see e.g.

(Feldman, 2006; Feldman and Mayes, 1999; Hoehl et al., 2020; Leong, Kalashnikova et al., 2017)). First, in Section 2 we discuss how entrainment might facilitate sensory processing (2.1) and outline the technical and methodological challenges involved in telling oscillatory entrainment apart from contingent responding (2.2). Then, in Section 3, we describe evidence that internal oscillators are present and active (indeed, in some cases more active) during early life.

In Section 4 we discuss the evidence that entrainment is present during early life, examining first behaviour (e.g. social interactional rhythms such as turn-taking behaviours) (4.1), then physiology (e.g. coregulation of autonomic arousal within infant-parent dyads) (4.2), then brain activity (e.g. neural entrainment to regular patterns and to complex patterns in speech) (4.3).

In Section 5 we consider the question: how is the emergence of volitional control characterised by changes in the inter-relationship between an individual and their day-to-day environment? First, we consider contingent responsiveness (5.1), and ask whether children who are more responsive to physical and social cues in their environment show better, or worse, volitional control. We argue that children with less volitional control should be more neurally and behaviourally sensitive to unattended objects in their environment. We also argue that the relationship between contingent responsiveness and volitional control may be moderated by the home environment - such that more sensitive individuals develop better volitional control only if they are raised in optimal home environments (e.g. more structured/ periodic as opposed to more chaotic/ unpredictable).

Next, we consider oscillatory entrainment. We differentiate between two types of oscillatory entrainment. First, we consider ‘top-down’ or ‘smart’ oscillators (5.2.1), driven by top-down processes of prediction and anticipation. ‘Smart’ oscillators are, we argue, likely to be later developing, and better able to process aperiodic stimuli. Next, we consider bottom-up’ or ‘dumb’ oscillators (5.2.2), driven by contingent evoked responsiveness to periodically spaced stimuli in the absence of any processes of prediction or anticipation. We argue that this type of entrainment may still play an important role in facilitating early learning, in cases where the environment is periodically structured.

Finally, we consider the relationship between how periodic, or predictable, a child’s early life environment is and their development of volitional control (Section 6). We discuss possible neural mechanisms through which making anticipations and predictions based on periodicities within the home environment might contribute to the development of volitional control.

## 2 Entrainment – potential benefits and measurement issues

### 2.1 Potential benefits of contingent responsiveness and oscillatory entrainment

Most approaches to studying how our brains and bodies respond to incoming information tend to take the same basic approach: they present different individual external stimuli, usually repeatedly, and examine contingent responsiveness to that stimulus. We can learn much from this approach – for example, by studying how contingent responsiveness varies as a function of our own pre-stimulus state (e.g. (Aston-Jones and Cohen, 2005; Wass, 2018)), and of the type of stimulus presented (e.g. (Graham and Jackson, 1970)). Contingent responding is also considered integral to regulatory functions, via corrective changes that allow us to maintain stability in the face of change (a process known as allostasis (Fiske and Maddi, 1961; Selye, 1951; Sterling, 2012)). Contingent responding to others also has coregulatory, social and communicative significance (Atzil et al., 2018, Feldman, 2007).

Other authors have, though, pointed out that this approach implicitly assumes that our brains and bodies respond purely reflexively and that background neural activity is noise (Ermentrout et al., 2008, Raichle, 2010). An increasing number of authors are emphasising instead that perception is not a passive reflexive process but rather a process of active prediction (Baek et al., 2020, Denham and Winkler, 2020, Ekman et al., 2017). Self-generated oscillatory rhythms are thought to play a vital role in this (Engel et al., 2001). A number of authors have suggested that, instead of merely reflexively responding to external changes when they occur, we may instead, or as well, start to predict the future occurrence of external changes before they occur - by detecting periodicities in the external stimuli, and adjusting our own patterns of internal periodic activity to match them (Ding and Simon, 2014, Lakatos et al., 2019, Poeppel and Assaneo, 2020, Thaut, 2013). This process is known as oscillatory entrainment.

**Fig. 1:**
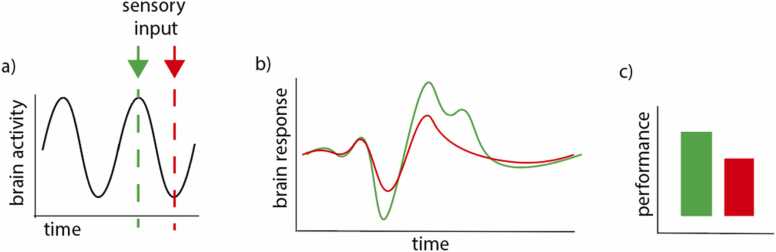
(a) Schematic illustrating the popular approach to understanding the relationship between neural phase and performance. Information that arrives at times of high neuronal excitability (shown red) evokes a larger phasic neural response (b) which in turn associates with increased behavioural sensitivity (c).

A number of authors have discussed potential benefits of oscillatory entrainment (Haegens and Golumbic, 2018, Lakatos et al., 2019, Poeppel and Assaneo, 2020). These discussions focus on how entraining internal oscillations to external oscillations can enable optimal processing of rhythmic stimuli. For example, sensitivity is thought to vary with phase, and aligning internal and external activity can help ensure that key sensory information is more likely to be processed during phases of high neuronal excitability (Busch et al., 2009, Calderone et al., 2014, Mathewson et al., 2009, VanRullen et al., 2011) (although see (Ruzzoli et al., 2019)) (see Fig. 1). (Of note, it remains unclear what the implications of this approach are for other studies that have shown *unexpected* targets to associate with superior learning, as these appear in some ways to be contradictory (Stahl and Feigenson, 2015).) Similar benefits of oscillatory entrainment have also been discussed in the context of interpersonal entrainment during social interaction (Feldman, 2007, Hoehl et al., 2020, Wass et al., 2020): interpersonal entrainment may make it easier to ensure, for example, that key items in a learning exchange are more likely to be delivered at a time when the learner is maximally sensitive (Wass et al., 2020).

As we shall see, though, despite the obvious potential benefits of oscillatory entrainment, demonstrating its existence is methodologically challenging (Meyer et al., 2020a, Rimmele et al., 2018). Many findings that are interpreted as providing evidence for oscillatory entrainment may instead arise from quite different mechanisms.

### 2.2 Measurement methods

All forms of entrainment – both contingent responding and oscillatory entrainment - can be formalised in two ways. First, sequential entrainment, which is a lagged relationship: ‘changes in A forward-predict changes in B’ (or vice versa). This is often tested using Granger-prediction (Granger, 1969). Sequential entrainment is asymmetric: it can be true that A predicts B without it being true that B predicts A. Second, concurrent entrainment: e.g. ‘when A is high, B is high’ or (for a negative relationship) ‘when A is high, B is low’. Concurrent entrainment is symmetric (A->B is equivalent to B->A). The popular term ‘synchrony’ technically refers to concurrent entrainment. But often, when it is used, it is driven by sequential entrainment (see e.g. (Feldman, 2007)). This is partly a function of windowing: a rapid sequential response can appear to cause concurrent entrainment if a larger time window is used (Haresign et al., 2021). But it is also a genuine phenomenon: if I look at you, and then you return my gaze, leading to mutual gaze, then sequential entrainment has caused concurrent entrainment.

#### 2.2.1 Contingent responsiveness and oscillatory entrainment

For present purposes, contingent responsiveness can be defined as evoked responses that involve changes in brain activity that arise directly from a new stimulus (Cohen, 2014). They are phase-locked, in the sense that the change that they bring to the phase of the neural activity is independent of the phase of the underlying neural activity at the moment that the stimulus was presented (Cohen, 2014, Makeig et al., 2004). Evoked physiological responses can include both increases and decreases in physiological activity (Wass, 2018).

Identifying oscillatory entrainment is more subtle. The definition of oscillatory entrainment is not controversial: oscillatory entrainment is observed when two oscillators become temporally aligned. This can take two forms. First, oscillatory entrainment of an individual to their physical environment, which is a one-way flow of information: the internal oscillator adapts themselves to the external oscillator. Second, oscillatory entrainment of one individual to another, which is, potentially (although not necessarily) a two-way flow of information: both oscillators adapt themselves to each other. The other factor that is uncontroversial is that, in neuroscience terms, these types of adaptation responses are characterised as *induced* responses – i.e. changes in underlying brain activity that are not driven purely by ‘additive’ brain responses involved in processing a new piece of information (Cohen, 2014, Makeig et al., 2004).

The subtlety comes, however, when we try to distinguish oscillatory entrainment from rhythmic contingent evoked responses. Many researchers define oscillatory entrainment through the phase alignment: when the oscillatory phases of two time series become aligned in time. This can be measured, for example, by calculating the Phase Locking Value (Lachaux et al., 1999). The challenge, however, is that evoked responses also affect the distritbution of phases with respect to the event (Makeig et al., 2002, Makeig et al., 2004) - meaning that, in cases where merely evoked responses are observed to periodically spaced stimuli, increased phase alignment would also be observed (Daume et al., 2021).

Another common mistake is to define oscillatory entrainment as the frequency adaptation of one oscillator to another – by claiming that, if (for example) changing the periodic stimulation rate from 4 Hz to 5 Hz reliably leads to equivalent changes in the rate of oscillatory activity in an attending brain, then oscillatory entrainment has occurred. But the example given in Section 1.2 shows, surely that this is too broad a definition: even if a brain is responding purely reflexively, showing evoked responses to each individual stimulus as they occur, then exactly that same pattern would be observed (Capilla et al., 2011, Haegens and Golumbic, 2018).

#### 2.2.2 Methods for distinguishing contingent responsiveness from oscillatory entrainment

How, then, can we differentiate the two? This debate is most well advanced in the context of entrainment to language, where it remains an ongoing debate whether neural tracking of the speech envelope reflects the resetting of ongoing oscillations in the auditory cortex (i.e., oscillatory entrainment) or additive brain responses to the physical attributes of the acoustic signal (i.e., contingent responsiveness) (Capilla et al., 2011, Doelling et al., 2019, Haegens, 2020, Meyer et al., 2020a, Meyer et al., 2020b, Rimmele et al., 2018).

One approach is to examine brain responses after the immediate cessation of a stimulus, or during a ‘missing beat’. An oscillatory entrainment model predicts that, after the cessation of a stimulus, neural activity would continue when the external stimulus was expected to occur, for at least the first ‘missing beat’ (Haegens, 2020, Meyer et al., 2020a). However, such a phenomenon might also be predicted based on a passive contingent responsiveness model, if we accept that a passive linear system with a damped response may still ‘decay’, or reverberate, after the cessation of a stimulus (see Section 5.2.2). A strict demonstration would, therefore, show oscillatory activity prior to rhythmic input, then coupling to the external oscillatory, and afterwards a return to the original state (Haegens and Golumbic, 2018).

Another approach, which to our knowledge has not been attempted, would be to present stimuli with a consistent average frequency, but jitter the individual stimuli by shifting the onset of each beat by a small margin. If the brain is contingently responding, there should be a neural response after each individual beat with an onset latency that would match the tempo that the beats were originally played. Oscillatory entrainment, in contrast, would predict that neural responses should align to the average frequency of the jittered beats.

Another common approach is to artificially induce ongoing neural oscillations using rhythmic sensory stimuli of transcranial brain stimulation (Thut et al., 2011). However, this approach, which also has inherent challenges (Lakatos et al., 2019), is not widely used with developmental populations. A variety of other, more indirect, methods have been used as well – such as computational modelling (Doelling et al., 2019) and an AutoRegressive Integrated Moving Average (ARIMA) model fitting to separate out stochastic and periodic components of the time series (Cohn and Tronick, 1988) (see Section 4.1).

Finally, other approaches have been taken by researchers working with different types of data – such as those looking at the timings of the onsets of vocalisations during a social exchange (see Section 4.1.2). For example, one study looked at whether consistent timings could be identified between vocalisation onsets in each partner considered separately (to identify whether each could be characterised as an oscillator). Then, they examined whether consistent timings could also be identified in the intervals between vocalisations in one monkey compared to a social partner (to identify whether they could be characterised as coupled anti-phase oscillators) (Takahashi et al., 2013).

## 3 Oscillators

In this section, we describe evidence that internal oscillators are present and active during early life, before we examine evidence of entrainment in Section 4.

Maintaining constancy of the internal environment is one of the operational principles of life (Gross, 1998). The most constant environment is, naturally, a fixed or steady state one; living organisms, however, must exchange matter, energy and information with the environment. Oscillatory activity, which is observed at all levels of biological organisation (Gamble et al., 2014, Goldbeter, 2018, Winfree, 2001) arises as a consequence of a trade-off between mechanisms that raise the level of systemic activity and those that control the inhibition of activity, allowing for non-equilibrium steady-state self-organisation (Decroly and Goldbeter, 1987, Goldbeter, 2018).

### 3.1 Physiology and behaviour

Our physiology has evolved to suit the planet we live on, driven by two primary oscillators: the yearly cycle (Miersch et al., 2013) and the day-night cycle (Silver and LeSauter, 2008) (although see (Ekirch, 2016)). From very early on, human newborns show cyclic organization of physiology and behaviour. Feeding and digestion, sleep and vigilance transitions, respiration cycles and vagally mediated heart rate variability at c 0.0.5 Hz; heart beats at (c.1–2 Hz); and the Mayer wave (oscillations of arterial pressure) at c 0.0.1 Hz (Cacioppo et al., 2000) are all examples of oscillators present from birth. These are thought to help newborns to maintain balance between mechanisms that raise the level of systemic activity and mechanisms that control the inhibition of activity (Feldman and Mayes, 1999).

Early foraging and attentional behaviours co-fluctuate with physiological changes (Hebb, 1949). Waking motor activity is inversely correlated with looking behaviour (Bacher and Robertson, 2001); both show oscillatory activity across the early months, which becomes less periodic and more stochastic through the first year of life (Robertson, 1985, Robertson, 1993, Robertson and Johnson, 2009).

Some research suggests that such cyclicity in the neonatal period and during the first months of life is related to more mature cognitive outcomes (Feldman and Mayes, 1999). For example, young infants whose attention patterns were more cyclic showed faster learning and discrimination (Feldman and Mayes, 1999) (see also (Feldman et al., 1996)). Greater sleep-wake cyclicity during early life also predicted superior emotion regulation during later development (Feldman et al., 2002), and increased physiological activity at the ~0.5 Hz range associated with better volitional attention control. This last finding has been shown in the context of vagally mediated heart rate changes (known as Respiratory Sinus Arrhythmia) (Thayer and Lane, 2000), which measures parasympathetic nervous system activity (Cacioppo et al., 2000); as well as for other physiological indices such as movement (Wass et al., 2018b). Taken together, these findings may illustrate that, particularly during early life, internal oscillators may help to maintain stable excitatory/inhibitory balances by organizing the information in regular periods of information intake and states of limitation on input (Turkewitz and Kenny, 1982).

Early vocal and social behaviours also correlate with periodic physiological fluctuations, in animals (Borjon et al., 2016; Zhang and Ghazanfar, 2016, Zhang and Ghazanfar, 2020) and in humans (Wass et al., 2021). Vocal development can be considered the outcome of interactions between the infant’s developing biological systems (the body and the nervous system) and their experience with caregivers (Ghazanfar and Zhang, 2016). Other social interactive rhythms involving crying, sucking and spontaneous movement may also be biologically based (Stratton, 1982), and their repetitive, temporally structured nature may provide the basis on which to form temporal expectancies that organize cognitive and affective experiences (Stern et al., 1975).

### 3.2 Neural activity and behaviour

Our brains show oscillatory activity at multiple scales, from slow-wave respiration-related oscillations at ~0.5 Hz (Tort et al., 2018) through to gamma band activity at > 50 Hz (Engel and Singer, 2001). Neuronal oscillations reflect the synchronisation of activity within and across nerve-cell populations (Fries, 2015). They are not an inadvertent epiphenomenon of how the brain operates (Buzsaki, 2006, Fries, 2015). Rather, oscillatory activity biases input selection, guiding cortical spiking activity (Fröhlich and McCormick, 2010) and temporally linking neurons into assemblies, playing a causal role in conscious perception and cognitive processing (Buzsaki, 2006, Helfrich and Knight, 2016). Research has suggested that the phase of neural activity at the time of stimulus presentation may relate systematically to the excitability of neural populations and the magnitude of event-related responses (Busch et al., 2009, Lakatos et al., 2008, Mathewson et al., 2009, VanRullen et al., 2011) (although see (Ruzzoli et al., 2019)); accordingly, sensory stimuli that are delivered during a high excitability oscillatory phase may be more likely to be detected and encoded than stimuli that arrive at an inhibitory oscillatory phase.

The dominant frequency of spontaneous brain activity seems to transition from theta (~5 Hz) to alpha (~10 Hz) with age (Cellier et al., 2021, Marshall et al., 2002) (see also (Schaworonkow and Voytek, 2021; Thierry, 2005)). Research that examined how oscillatory activity co-fluctuates with volitional control has primarily suggested that volitional control associates with phasic increases in theta (2–6 Hz) and decreases in alpha (6–9 Hz) (Begus and Bonawitz, 2020, Orekhova et al., 1999, Orekhova et al., 2001, Orekhova et al., 2006, Wass et al., 2018, Xie et al., 2018, Xie et al., 2019). Other studies have associated more oscillatory neural activity in the theta range, with better short- and long-term cognitive outcomes (Braithwaite et al., 2020; Jones et al., 2020).

Recent research has also suggested that, in contrast to the abundance of other types of oscillator described above, endogenously generated neural activity may in fact be relatively less oscillatory and more aperiodic during early development (Schaworonkow and Voytek, 2021) (see also (van Ede et al., 2018)). This is important because even when no oscillation is present, spectral analyses will show power within a frequency band driven entirely by the aperiodic signal, and not by any oscillatory activity. Thus, when the aperiodic signal is overlooked, one cannot say with certainty whether the band-specific power changes seen in development are driven by changes in oscillatory bursts, the aperiodic signal, or both (Schaworonkow and Voytek, 2021). As of yet, no study has managed to disentangle which features are truly changing with development (the aperiodic signal, and/or oscillatory burst amplitude and oscillatory frequency).

### 3.3 Self-generated rhythms

In adults, sensory sensitivity fluctuates rhythmically. For example, the ability to detect stimuli that are at the threshold of an individual’s sensory sensitivity fluctuates at approximately 8 Hz (Busch et al., 2009, Landau et al., 2015, Landau and Fries, 2012, VanRullen, 2016). Many authors have noted that these fluctuations in sensory sensitivity appear to match with the tempo of many self-generated rhythms, such as eye movements during visual foraging (Otero-Millan et al., 2008) – and, in animals, periodic whisking or sniffing (Kleinfeld et al., 2016) (for reviews, see (Haegens and Golumbic, 2018; Lakatos et al., 2019)) – as if the tempi at which we spontaneously sample from the environment have adapted to suit our own endogenous fluctuations in sensory sensitivity. (Although it is important to remember that these types of ‘arguments by coincidence’ are unfalsifiable (Rose and Rose, 2010)). Others have, similarly, noted that our spontaneous communicative behaviours, such as rhythmic fluctuations in speech, also coincide with our fluctuations in sensory sensitivity (Poeppel and Assaneo, 2020) – as if the tempi at which we spontaneously communicate have also adapted to suit our partners’ fluctuations in sensory sensitivity.

No work has, to our knowledge, investigated fluctuations in sensory sensitivity during early development. The speeds at which we spontaneously sample from the environment have, however, been measured. Eye movements during visual foraging are slower in infants compared with adults (Bronson, 1990, Bronson, 1994, Wass and Smith, 2014), as are fluctuations in overt (Feldman and Mayes, 1999) and covert (Robertson et al., 2012) attention on the second scale (e.g. looking to pictures during habituation). The dominant speed of amplitude fluctuations in infant-directed speech is also slower than in adult-directed speech (Leong et al., 2017, Leong and Goswami, 2015, Narayan and McDermott, 2016) – a preference which emerges independent of experience (Masataka, 1999). It may be that these slower-tempi sensory sampling and communicative behaviours are related to the fact that spontaneous fluctuations, which drive fluctuations in sensory sensitivity are slower in infants, too. Again, though, this is an argument by coincidence.

## 4 Evidence for entrainment

### 4.1 Behavioural entrainment

#### 4.1.1 Entrainment to the physical environment

The only research to have examined early behavioural entrainment to the physical environment has looked at entrainment to music. For example, one study suggested that 5–24-month-old infants show more spontaneous movement when listening to music or to a simple rhythm derived from the music than when hearing recorded speech, and moved faster when the tempo of the auditory rhythm was faster (Zentner and Eerola, 2010); however, more direct measures of entrainment were not taken. Another study asked 2- to 4-year-old children to move along with familiar music presented both at the original and at modified tempi and recorded their head movements. Autocorrelation analyses revealed evidence of periodic movement but little adaptation to tempo changes in the music (Eerola et al., 2006). Another study with 5- to 11-month-old infants showed evidence of improved tempo matching with age (Rocha et al., 2021), although entrainment was not directly measured (see also (Kirschner and Tomasello, 2009)). Taken together, these findings seem to suggest that, from very early on, human infants might entrain to the physical environment. However, more direct measures of entrainment are needed.

#### 4.1.2 Entrainment to the social environment

Ample evidence suggests that, when dyads interact together, multiple aspects of their behaviour start to mimic one another (Condon and Sander, 1974, Feldman, 2007, Schneirla, 1946). This is true across facial expressions, linguistic expressions, manual gestures, and noncommunicative postures techniques (Chow et al., 2010, Louwerse et al., 2012, Schmidt et al., 2014, Shockley et al., 2002). Turn-taking behaviours also develop during verbal and nonverbal communications, in animals (Takahashi et al., 2013, Takahashi et al., 2016) as well as humans (Fusaroli et al., 2014, Hilbrink et al., 2015). Behavioural mimicry appears to increase with age: for example, Feldman and colleagues used cross-correlations to measure how the strength of associations in facial affect between infants and parents varies over time. They found that both infant->mother and mother->infant influences increased from 3 months to 9 months (Feldman et al., 1996). With increasing age, other factors such as social context increasingly mediate and modulate mimicry (van Schaik and Hunnius, 2016).

A number of authors have directly considered whether this behavioural entrainment is driven by contingent responsiveness or oscillatory entrainment. Lester and colleagues identified oscillatory components (between.02 and 0.1 Hz) in changes in facial affect during an infant-parent exchange, and computed coherence to identify significant correlations between the two time series at each frequency (Lester et al., 1985). However, as we pointed out in Section 2.2.1, such a finding would also arise merely from contingent responsiveness, without any oscillatory entrainment.

Another paper took a different approach, using ARIMA models to separate changes in facial affect during an infant-parent tabletop interaction into stochastic and oscillatory components; separately, they measured infant-parent bidirectional influences using cross-correlations. They found that at no age was the occurrence of oscillations in the parent’s or infant’s behaviour related to the achievement of bidirectional influence – suggesting that the bidirectional influences they observed (infant influencing parent, and parent influencing infant) were brought about through contingent responding rather than oscillatory entrainment (Cohn and Tronick, 1988).

A larger body of research has examined turn-taking behaviours in conversation, following a suggestion that these may be instantiated through oscillatory entrainment in the brains of speaker and listener (Wilson and Wilson, 2005). Although some findings have not been consistent with this (O’Dell et al., 2012), others have. For example, a study on vocal turn-taking in monkeys identified consistent inter-call intervals between calls in monkeys (suggesting oscillators), along with consistent inter-call intervals between one monkey’s call timings with respect to their paired social partner (suggesting coupled anti-phase oscillators) (Takahashi et al., 2013). The same study also found entrainment, in the sense that if one speeds up or slows down their call timing, the other will do so as well (Takahashi et al., 2013).

Although there are some suggestions that turn-taking is present even as young as 2–4-days (Dominguez et al., 2016) (see also (Gratier et al., 2015; Jasnow and Feldstein, 1986)), others suggest that turn-taking behaviours increase over time, in humans (Elias and Broerse, 1996, Stern et al., 1975) and monkeys (Takahashi et al., 2016). This is consistent with the evidence for bidirectional influences in facial affect described above.

Overall, these findings suggest that mimicry and turn-taking behaviours develop across multiple modalities early in development, but it is unclear whether these are driven by contingent responsiveness or oscillatory entrainment. The strongest evidence for oscillatory entrainment is for vocal turn-taking behaviours.

### 4.2 Physiological entrainment

#### 4.2.1 Entrainment to the physical environment

There is evidence that, in adults, autonomic features such as heart rate, blood pressure and respiration rate are influenced by the physical environment – such as while listening to fast-paced music (Trost et al., 2017). However, the most commonly studied measures (e.g. heart rate, respiration frequency) can only show adaptation up to a point (Trost et al., 2017). Although the relevance of physiological entrainment to music in infancy has been discussed (Hoehl et al., 2020, Markova et al., 2019) it has not to our knowledge been examined. Another study used cross-correlations to show that increases in noise in the home environment associates with increases in autonomic arousal, although oscillatory structures were not examined (Wass, Smith, Daubney et al., 2019). Taken together, these findings suggest our physiological systems respond contigently to sounds in our environment, but no evidence exists for oscillatory entrainment.

#### 4.2.2 Entrainment to the social environment

There is also evidence for physiological entrainment to the social environment – although this is not direct synchronisation, as infants’ dominant heart beat and respiration rate, for example, tends to be ~x1.5 that of adults (see (Noujaim et al., 2004)). At times during a parent-infant free play interaction when one partner’s heart rate increases, the other partners’ does, too (Feldman et al., 2011). Subsequent work using a similar method identified transient increases in the degree of mutual influence between child and parent autonomic arousal following negative but not positive affect vocalisations (Wass et al., 2021, Wass et al., 2019). When a parent shows greater contingent responsiveness by increasing their own autonomic arousal to match their child’s, the child calms more quickly (Wass, Smith, Clackson et al., 2019).

The degree to which physiological entrainment is observed is thought to be affected by other factors, such as the pre-existing relationship between the interaction partners, social affiliation (Hoehl et al., 2020, Konvalinka et al., 2011, Schirmer et al., 2016) and even mental wellbeing. For example, (Smith et al., 2021) found excessive contingent responsiveness in anxious parents over-reacting to small-scale fluctuations in their child (Smith et al., 2021) (see also (Feldman et al., 2009)).

Overall, no research to our knowledge has examined whether physiological coupling is driven by contingent responsiveness or oscillatory entrainment. Although it would be possible to study this (see e.g. (Y. S. Zhang and Ghazanfar, 2016, Zhang and Ghazanfar, 2020) for analogous approaches), these methods have not yet been used to study early development.

### 4.3 Neural entrainment

#### 4.3.1 Entrainment to the physical environment

A body of research has measured early life entrainment to ‘pure’ periodic stimuli (sometimes known as steady state evoked potentials, or frequency tagging), both in the visual (e.g. (Köster et al., 2019; Wieser et al., 2016)) and auditory (Cirelli et al., 2016) domains (see (Norcia et al., 2015) for review). Strong neural responses are observed in infants contingent on the frequency of stimulation; in some cases these also show sensitivity to higher-order musical structure, such as meter (Cirelli et al., 2016, Winkler et al., 2009).

One way to determine whether these represent contingent responding or oscillatory entrainment is by presenting ‘pure’ periodic stimuli interspersed with ‘missing beats’, as described in section 3.2.2. Some authors have taken this approach, and found that even newborn infants show, in some settings, neural responses to a missing beat presented in the context of music (Winkler et al., 2009). Similar phenomena have been widely observed in adults, albeit with some caveats (Denham and Winkler, 2020). This is consistent with oscillatory neural entrainment – although see the caveats in Section 2.1 (Trost et al., 2017).

Other approaches have been used to look at neural responses in dynamic, non-repetitive situations more similar to those encountered in the real world. Several studies have used a temporal response function, in which regression/Granger-predictive techniques are used to predict the neural signal based on stimulus features (Jessen et al., 2021). For example, one study quantified luminance, motion and the auditory speech envelope in a 5-minute cartoon and found that infant EEG was significantly predicted by both video motion and the auditory speech envelope (Jessen et al., 2019). Two other studies have used the same approach to identify associations between infants’ EEG activity and the auditory envelope of speech (Attaheri et al., 2021; Kalashnikova et al., 2018). Although evidence of entrainment, this is a regression-based measure, and the oscillatory structure of either time series was not directly examined.

#### 4.3.2 Entrainment to the social environment

Recent research, building on animal (Kingsbury et al., 2019; W. Zhang and Yartsev, 2019) and adult (Liu et al., 2018) studies, has identified interpersonal neural entrainment between infants and adults during social interaction (see (Markova et al., 2019, Wass et al., 2020) for recent reviews). This has been shown at multiple time-scales, from fluctuations over the second scale using fNIRS (Nguyen et al., 2020, Nguyen et al., 2021, Nguyen et al., 2020, Piazza et al., 2020) through to fluctuations over the sub-second scale using EEG (Leong et al., 2017, Santamaria et al., 2020). Other studies have also shown direct patterns of entrainment between brain and behaviour across a parent-child dyad (Wass et al., 2018a).

The methods used to show this include: generalised partial directed coherence, which involves predicting neural activity in the frequency domain in one individual based on neural activity in their social partner (Leong, Byrne et al., 2017); phase-locking value (see section 3.2.1) (Santamaria et al., 2020); and wavelet transform coherence, which measures both concurrent and sequential synchrony of two signals in the time-frequency plane (Nguyen et al., 2020, Nguyen et al., 2021, Nguyen et al., 2020, Piazza et al., 2020). The question of whether this entrainment is driven by contingent evoked responses happening in two brains concurrently, or by oscillatory entrainment, remains to be explored (Wass et al., 2020).

## 5 Associations between early-life entrainment and volitional control

### 5.1 Contingent responsiveness and early life volitional control

#### 5.1.1 Physical environment

A number of infant studies have shown that greater physiological responsiveness (relative both to *externally* defined events (experimenter-defined stimulus presentations) and to *internally* defined events (infants’ looks to and away from the target)) associate with superior learning and reduced distractibility (reviewed (Richards, 2010, Richards, 2011)). For example, infants can better recognise visually presented stimuli during heart rate decelerations (Frick and Richards, 2001, Richards, 1997), and are less distractible during heart rate decelerations (Casey and Richards, 1988, Lansink and Richards, 1997). EEG studies have suggested that larger neural contingent responses are elicited during heart rate decelerations (de Haan, 2008, Richards, 2003). Based on these findings, we might conclude that phasic relationships exist, such that increases in contingent responsiveness associate with increases in the volitional control of attention towards the attended stimulus (Wass, 2018). However, no research has examined this from the perspective of individual differences: whether individuals who show greater contingent responsiveness also show superior volitional control.

Evidence from adult studies suggests that directing attention towards a particular object or stimulus feature leads to larger contingent evoked physiological (Frith and Allen, 1983) and neural responses towards that particular object or stimulus feature (Corbetta et al., 1991, Desimone and Duncan, 1995, Kok et al., 2012, Picton and Hillyard, 1974), along with smaller contingent evoked responses to unattended objects or features. For example, reduced stimulus-related ERPs are reported during mind-wandering (Baird et al., 2014). Based on these findings, we might expect that children with greater capacity to direct attention towards a particular object should show greater contingent responsiveness towards that object and, at the same time, show reduced responsiveness to unattended objects (or streams of information). The magnitude of this attentional effect (attended – enhanced/ unattended – suppressed) should associate positively with volitional control.

For unattended stimuli, though, we predict the opposite: that reduced volitional control should associate with reduced suppression of responses to unattended / distracting information, and that the differences in responsiveness to attended vs unattended stimuli will be smaller (see e.g. (Kok et al., 2012; Stevens et al., 2009)).

When considering physiological responsiveness, an additional challenge is that one system (the Autonomic Nervous System) is responsible for responding both to positive, attention-eliciting stimuli and to negative, aversive stimuli (Kok et al., 2012, Stevens et al., 2009). Because of this, there are suggestions that repeatedly evoking a physiological response in aversive contexts (such as children raised in less supportive environments) may lead to that system becoming desensitised and less capable of supporting attention in both negative but also positive contexts (Wass, 2018, Wass et al., 2019). Differential Susceptibility Theory holds that more sensitive individuals (i.e. those with a tendency to show greater contingent responsiveness overall) may show superior long-term outcomes in positive environments, but worse long-term outcomes in negative environments (Belsky et al., 2007, Boyce and Ellis, 2005, Obradovic, 2016, Wass, 2018). Thus, the relationship between contingent physiological responsiveness and volitional control may be moderated by the home environment.

#### 5.1.2 Social environment

The entrainment of an individual to their physical environment is uni-directional (see Section 2): the individual entrains to their environment, but not vice versa. The entrainment of an individual to their social environment (for example in infant-parent interactions) can, however, be bi-directional: we can consider how a parent entrains to their child, and how a child entrains to their parent, as two separate measures. We ought, therefore, to generate two separate sets of predictions. First, we can consider how the responsiveness of a parent to their child relates to long-term child outcomes. Second, we can consider how the responsiveness of a child to their parent relates to long-term child outcomes. Here, for reasons of space, we concentrate on the second of these two questions. (These other papers have addressed the first question (Fay‐Stammbach et al., 2014; Mason, 2018; Vernon-Feagans et al., 2016)).

Although a number of studies have reported that children become more responsive to their parents through early life (e.g. to changes in facial affect during joint play (Feldman et al., 1996) and to turn-taking behaviours in conversation (Elias and Broerse, 1996; Stern et al., 1975)) only one study has, to our knowledge, directly examined how children’ responsiveness relates to later volitional control. This study looked at the relationship between bidirectional influences in facial affect during tabletop play at 3 and 9 months and self-control at 2 years. 9-month-old children who were more responsive showed superior self-control at 2 years after temperament, IQ, and maternal style were partialled out; the same relationship was not observed at 3 months (Feldman et al., 1999). In addition, infant difficult temperament moderated the relationship between mutual synchrony at 9 months and self-control at 2 years, such that stronger relations between mutual synchrony and self-control were found in infants with difficult temperament (Feldman and Mayes, 1999) (see also (Oshri et al., 2021)).

Based on this, we would expect that contingent responsiveness to the social environment should associate positively with volitional control. Note, however, that infants’ responsiveness to social cues has only been assessed through tabletop, face-to-face interactions, in which only one social partner is present. In more complex settings, in which multiple social partners are present and not all social signals are directly attended to, we might also make the same prediction as discussed in Section 5.1.1: that increased volitional control should associate with increased responsiveness to attended social cues, and decreased responsiveness to unattended social cues. And we might also make the same prediction as in Section 5.1.1 for how the environment moderates this relationship: that more sensitive individuals should develop better volitional control only if they are raised in optimal home environments.

### 5.2 Oscillatory entrainment and early life volitional control

Our discussion of the potential benefits of oscillatory entrainment started from the difference between ‘traditional’ approaches, that view perception as a purely passive, reflexive, stimulus-driven process, and more recent approaches that instead emphasise the role of active prediction in perception (e.g. (Engel et al., 2001)). This distinction goes to the heart of the distinction between contingent responsiveness and oscillatory entrainment that forms the backbone of this article.

**Fig. 2:**
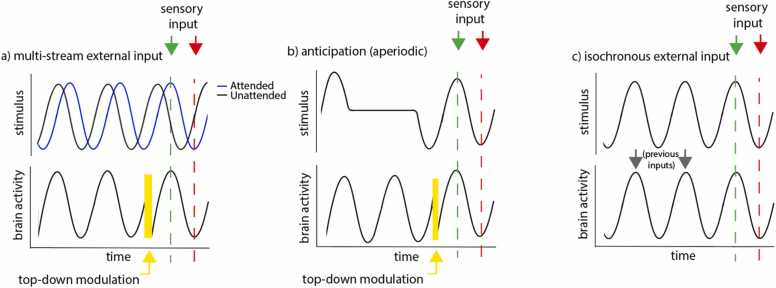
Illustrating three different mechanisms that might underlie the relationship between the timing of sensory input and phase-related changes in neural excitability. a) a classical ‘top-down’ entrainment model, top-down modulation changes the phase of the underlying neuronal activity to align the neuronal oscillatory activity with the attended-to sensory stream; b) top-down modulation in anticipation to aperiodic stimuli; c) an illustration of the ‘dumb’ oscillator mechanism described in Section 5.2.2. An isochronous auditory cue facilitates perceptual processing of a subsequent target in cases where the target in presented in-phase with the previous auditory cue.

In this section, we discuss these approaches to understanding oscillatory entrainment as the product of top-down/modulatory input (Section 5.2.1 (‘Smart’ oscillators) (see also Fig. 2)). We then go on to make an additional point, arguing that some of the facillitatory effects of oscillatory entrainment on stimulus processing might also be achievable purely via entrainment driven by contingent responses, without any active, predictive processes being involved at all. We discuss this in Section 5.2.2 (‘Dumb oscillators’) (see Fig. 2c). Naturally, the relationship of each of these two forms to volitional control is markedly different.

#### 5.2.1 ‘Smart’ oscillators

In adult cognitive neuroscience evidence for top-down modulation of oscillatory entrainment has been shown mainly using paradigms that are unavailable to infant researchers: those that ask a participant to voluntarily direct their attention in a particular way. Although different variants of this have been used (e.g. (Bonnefond and Jensen, 2012)), the most popular are those using versions of the ‘cocktail party effect’, in which multiple sensory streams are presented (either in the same, or different, modalities) and the participant is asked to direct attention to one of them. In adults, greater low-frequency neural entrainment is observed to the attended-to stream (e.g. (Golumbic et al., 2013; Lakatos et al., 2008, Lakatos et al., 2008)). Although the same phenomenon would also be observed even in the absence of oscillatory entrainment (because, as discussed in Section 5.1, evoked responses to attended-to stimuli are larger), phase-shifts in entrainment were also observed which cannot be explained by simple bottom-up representations of stimulus properties (Golumbic et al., 2013, Lakatos et al., 2013) (see Fig. 2a). Although still controversial, this is generally held to be top-down modulated (Haegens and Golumbic, 2018, Park et al., 2015, Poeppel and Assaneo, 2020). For example, one study assessed the connectivity between the auditory cortex and other areas while participants listened to natural (intelligible) speech and backwards (unintelligible) speech (Park et al., 2015). Their data suggested that oscillatory activity generated in the left inferior frontal and precentral gyri modulated the phase of low-frequency activity in auditory regions, significantly more in the intelligible than the unintelligible condition (Park et al., 2015).

Other research has suggested that the mechanisms which drive top-down modulation of oscillatory entrainment to periodic stimuli may be quite similar to those involved in generating predictions for aperiodic stimuli, where it has also been shown that prediction facilitates sensory processing (Haegens and Golumbic, 2018, Rimmele et al., 2018) (see Fig. 2b). For example, one study shows that periodic and aperiodic temporal predictions both induce similar phase alignment of low-frequency activity before an expected target (Breska and Deouell, 2017, Daume et al., 2021). These observations contrast with more traditional entrainment models which hypothesize that reducing stimulus periodicity should result in reduced phase alignment (Obleser and Kayser, 2019, Rimmele et al., 2018).

Some behavioural evidence has used simplified versions of the ‘cocktail party effect’ – for example, looking at infants’ ability to recognise their name amongst multiple speech streams. These studies suggest that childrens’ capacity for selectively attending to one amongst multiple speech streams is much more rudimentary in infants (Newman, 2005) and children (Sussman and Steinschneider, 2009) than adults. Childrens’ cortical tracking of speech in noise is detectable by 6–9 years, but is weaker, particularly at higher frequencies (>1 Hz) and more affected by multitalker background level (Vander Ghinst et al., 2019). Compared with adults, young children’s behavioural sensitivity is also less facilitated by predictions and anticipations (Addyman et al., 2014).

No study has directly investigated how oscillatory entrainment driven by top-down modulation develops through infancy and childhood. The evidence reviewed above, however, is consistent with starting expectation (Section 1.1) that the capacity for oscillatory entrainment driven by top-down modulation (as shown in adults e.g. by (Park et al., 2015)) should be relatively rudimentary at birth, and develop through childhood.

#### 5.2.2 ‘Dumb’ oscillators

If a stimulus creates an evoked response, and if that stimulus is presented at regular time intervals, then these evoked responses will also show a regular periodic structure. In Section 4 we reviewed several sources of evidence for behavioural, physiological and neural entrainment that were, we argued, either possibly or probably attributable purely to this mechanism – i.e. that showed evidence for phase entrainment, or for the frequency adaptation of one system to another (see Section 2.2.1), but which may have been attributable entirely to contingent responsiveness.

The main potential benefit of oscillatory entrainment, as we discussed in Section 2.1, is that oscillatory entrainment may facilitate sensory processing because aligning internal and external activity can help ensure that key sensory information arrives during times when sensory processing is at its greatest. Because larger amplitude brain responses are observed for environmental events that occur in-phase with brain activity, neural responsiveness may be selectively increased to periodicities within the early-life environment. This would produce effects that are similar to directed attention, which associates with increases neural responsiveness to the attended-to stream. But in this case the selectively increased neural responsiveness would be driven by external properties (periodicities) within the environment. Importantly, this benefit would also be present whether the alignment of internal activity is driven by top-down modulation (as is more traditionally described), or arrived at purely in a bottom-up, evoked, sensory driven manner (see Fig. 2c) (Haegens and Golumbic, 2018, Sauseng et al., 2007, Schroeder et al., 2008).

Of course, these benefits would only continue after the cessation of the external sensory stimulation for targets presented in phase with it, and for as long as the periodic activity induced by the external stimulus continued to ‘reverberate’ internally, as a damped response – which would of course vary between different systems (behaviour, physiology, neural activity) (Gulbinaite et al., 2016, Lerousseau et al., 2021, van Bree et al., 2021). Similar to the literature on ‘smart’ entrainment, it might also be that stronger effects are identified where the stimulation frequency is closer to the dominant frequency of the participant’s endogenous frequency (known as the Arnold tongue) (Notbohm et al., 2016). Finally, they would differ between infants/children and adults. Although it is unclear whether this is a property of ‘smart’ or ‘dumb’ oscillatory activity, extensive research with adults has shown, for example, that rhythmic auditory cues can facilitate the sensory processing of a subsequent target even for several cycles after cue offset (M. R. Jones et al., 1981, Jones et al., 2002). Similar findings have been shown in multiple other contexts and sensory domains (although see (Haegens and Golumbic, 2018) for inconsistent findings and important caveats). No research has investigated this in developmental populations.

Although the example given above examines sensory processing relative to neural oscillations on the millisecond-level scale, there is no reason why similar mechanisms would not also apply at the other temporal scales we discussed in 2, 3. For example, similar mechanisms may explain how coupled oscillatory dynamics can emerge during turn-taking social exchanges even in marmoset monkeys (Takahashi et al., 2013).

Although both may have benefits for learning, the difference between ‘dumb’ and ‘smart’ oscillatory entrainment would show most markedly in the degree to which differences in oscillatory entrainment (characterised by phase shifts that are not purely attributable to bottom-up stimulus properties) are observed between attended vs unattended stimuli (see Section 7.2 for concrete predictions). For ‘smart’ entrainment, increased oscillatory entrainment would be expected for attended vs unattended streams. For ‘dumb’ entrainment, it would be driven more by the salience of the stimulus, and in cases where the stimulus was strictly (as opposed to approximately) periodically spaced. Another important difference is that the benefits of ‘dumb’ oscillatory entrainment would be limited to stimuli that are strictly periodic. Whereas if ‘smart’ entrainment is driven by similar mechanisms for both periodic and aperiodic stimuli (Obleser and Kayser, 2019, Rimmele et al., 2018), then it should be robust to aperiodic stimuli.

‘Dumb’ entrainment might, putatitvely, be an early-developing mechanism – in contrast to ‘smart’ entrainment which, as argued above, is likely to develop later. This might, then, offer a mechanism for why strictly isochronous stimuli facilitate learning during early as well as later development: because the periodicities in the stimulus allow neural responsiveness to be selectively increased to that stimulus, relative to other aperiodic stimuli present in the environment, producing effects which are similar to directed attention, but driven purely by external properties (periodicities) of the stimulus. Ample evidence suggests that our early environments tend to be more periodic, across multiple temporal scales (de Barbaro and Fausey, 2021, Warlaumont et al., 2022). For example, early language-based interactions often rely more heavily on nursery rhymes and rhythmic singing (Britto et al., 2002, Markova et al., 2019, Mendoza and Fausey, 2021); sub-second-level amplitude modulations patterns in infant-directed speech are more periodic compared to adult-directed speech (Goswami, 2018, Hilton et al., 2021, Leong et al., 2017, Leong and Goswami, 2015, Leong and Goswami, 2015); attentional foraging patterns are more profoundly periodic during early life (Robertson, 1985, Robertson, 1993); and daytime routines are often more consistent during early childhood (Spagnola and Fiese, 2007). And as we noted above (in Section 3), some authors have also reported that infants who show stronger periodicities in their patterns of self-generated behaviours show superior attention and learning (Feldman et al., 1996, Feldman and Mayes, 1999, Frensch et al., 2011).

## 6 Aperiodic early life environments and volitional control

Some children’s early life environment is more periodic than others’ (Evans and Wachs, 2010, Glynn et al., 2021, Papadimitriou et al., 2021), across a variety of scales – from daily routines (Roche and Ghazarian, 2012) and sleep-wake cycles (Feldman, 2006, Weisman et al., 2011) through to interactional rhythms (e.g. turn-taking behaviours in early social exchanges) (Feldman, 2007) and early language exposure (Narayan and McDermott, 2016).

Some indirect evidence suggests that more periodic early life environments associate with better volitional control. For example, questionnaire ratings of household chaos predict long-term child outcomes, including effortful control (Evans and Wachs, 2010, Marsh et al., 2020, Martin et al., 2011) – although other factors such as noise (Wass, Smith, Daubney et al., 2019) or parental responsiveness (Vernon-Feagans et al., 2016) may also mediate this. Unpredictable maternal sensory signals (visual, auditory and tactile) predict worse long-term cognitive outcomes (Davis et al., 2017) and increased risk of later psychopathology (Baram et al., 2012, Glynn and Baram, 2019, Molet et al., 2016). The same relationship has also been shown in animal models (Davis et al., 2017), which partially precludes the possibility that the correlation is due to some unobserved third factor, such as sociodemographic factors (Evans et al., 2005),. None of these studies have, however, directly sampled from the home environment, preferring instead to use self-report questionnaires or observations of lab-based interactions.

In Section 5.2.2 we discussed the possibility that isochronous environmental stimulation might directly facilitate perceptual processing for subsequent targets presented in-phase with that previous environmental stimulation. Beyond this, however, is it possible that more periodic environments might directly facilitate the development of predictions and anticipations, and through this the development of top-down mediated oscillatory entrainment and volitional control? No research to our knowledge has examined this.

Investigating this question fully would likely require animal research, in which the environment can be strictly controlled, in combination with human research which quantifies temporal regularities in the home environment. One prediction to be investigated (based on (Daume et al., 2021, Rimmele et al., 2018)) is that top-down modulated phase entrainment might develop through repeated exposure to periodic stimuli, but that these long-term facilitatory effects would also help in processing aperiodic stimuli as well (see Section 5.2.2).

## 7 Conclusion

### 7.1 Summary

In this article we have examined the question: how is the emergence of volitional control characterised by changes in the relationship between an individual and the sights and sounds in their everyday environment? We have differentiated two ways of quantifying this relationship: contingent responding (i.e. evoked responses within an individual to changes in their outside environment) and oscillatory entrainment (i.e. already periodic activity patterns within an individual becoming coupled, or temporally aligned, with oscillations in the environment).

The evidence we reviewed has suggested that oscillators are ubiquitous during early development at multiple levels. Early foraging and attention behaviours, as well as early vocal and social behaviours, both co-fluctuate with periodic physiological changes (3.1, 3.3). Although brain oscillations during early development are less well studied, oscillations within particular frequency ranges are thought to co-fluctuate with attention (Section 3.2), and it seems likely that perceptual sensitivity varies with oscillatory phase, as has been shown in adults. Children whose spontaneous foraging behaviours are more periodic show better attention and learning (Section 3.1).

We also reviewed evidence suggesting that children show behavioural, physiological and neural entrainment to their physical environment (e.g. noises, music) and to their social environment (e.g. facial expressions, communicative gestures, vocalisations etc) (Section 4). And we reviewed evidence for direct interpersonal entrainment of brain activity (Section 4.3.2) and physiology (Section 4.2.2). Overall we concluded that, although ample evidence suggests that infants show contingent evoked responsiveness, convincing evidence for oscillatory entrainment is generally lacking.

Evidence for top-down modulation of oscillatory entrainment as a mechanism of attentional selection in early development is also lacking (Section 5.2.1), consistent with behavioural evidence that this capacity is trace during early development. However, we also argued that, in cases where the environmental stimulus is strictly periodic, some of the facilitatory effects of oscillatory entrainment on perceptual sensitivity may still be obtained even purely from contingent responsiveness – which might explain the importance of periodic environmental rhythms during early learning (Section 5.2.2). Because larger amplitude brain responses are observed for environmental events that occur in-phase with brain activity, neural responsiveness may be selectively increased to periodicities within the early-life environment. This would produce effects that are similar to directed attention, which associates with increases neural responsiveness to the attended-to stream - but in this case the selectively increased neural responsiveness would be driven by external properties (periodicities) within the environment. Finally, we discussed the potential role of oscillatory entrainment as a mechanism that might mediate the relationships observed between how periodic a child’s environment is and their long-term development of volitional control (Section 6).

### 7.2 Predictions

From the discussion in 5.2.1, 5.2.2 we have derived the following predictions:

• Prediction 1: an isochronous auditory cue should facilitate perceptual processing of a subsequent target.
  And if ‘dumb’ entrainment occurs then during early infancy:
• Prediction 2: the effect of Prediction 1 should be stronger when the auditory cues are more salient.
• Prediction 3: Prediction 1 should only be observed for targets presented in phase with the previous rhythmic cues, and for as long after the cessation of the cue as periodic activity induced by it continues to ‘reverberate’ as a damped response.
• Prediction 4: Prediction 1 should not be observed more strongly when the target is attended vs non-attended.
• Prediction 5: Prediction 1 should not be observed when non-isochronous (e.g. jittered) cues are presented.

As ’smart’ entrainment develops then during later infancy:

• Prediction 6: Prediction 1 should also be observed in situations where the auditory cue is predictable but not strictly isochronous.
• Prediction 7: Prediction 1 should be observed more strongly when the target is attended vs non-attended.
• Prediction 8: Prediction 1 should be observed more strongly when the cue is presented at a rate which is close to the participant’s dominant frequency of intrinsic oscillatory activity (the Arnold tongue).

### 7.3 The ghost learning to drive the machine

We started this article by asking: how does the ‘ghost’ (the active, volitional component of cognition) learn to ‘drive the machine’? Specificially, how does the emergence of volitional control affect the inter-relationship between activity within an individual and the environment in which that individual is located?

Relatively little empirical work so far has been conducted in this area. The methodological challenges in differentiating between different mechanisms of entrainment remain formidable. Nevertheless, we have argued that some sort of picture is starting to emerge.

When the ghost takes charge of the machine, (s)he isn’t taking charge of an inanimate hunk of metal, that is lying silent on the tarmac until (s)he turns the ignition. Rather, the car is already moving as (s)he starts to take charge – reflexively bumping along, driven by rhythms in the outside environment. (S)he learns to drive by listening to rhythms that are already present in the machine, and spotting patterns in how it functions. Over time, as these patterns become clear, (s)he gains the ability to nudge, and to alter the machine’s future path.

In some ways, the relationship is like a rally driver driving over sand dunes. It is a bumpy ride, and initially the driver has next to no control, getting thrown here and there by the sand dunes that come their way. Over time, though, (s)he gradually learns to predict when the peaks and troughs will occur; and, by using this information, she learns to judge when to nudge the pedals or to turn the steering wheel slightly, so as to start to gain control.

## Declaration of Competing Interest

The authors declare the following financial interests/personal relationships which may be considered as potential competing interests: Sam Wass reports financial support was provided by European Research Council.

## Data Availability

This article is a review article and so does not contain any novel empirical data that are being published for the first time.
